# Disparities in Outcomes in Latino Subpopulations with Localized Prostate Cancer Undergoing Radical Prostatectomy: A Population-Based Analysis

**DOI:** 10.3390/cancers18061035

**Published:** 2026-03-23

**Authors:** Salvador Jaime-Casas, Regina Barragan-Carrillo, Anjaney Kothari, Wesley Yip, Oluwatimilehin Okunowo, Ahmad Imam, Daniel J. Lama, Alexander Chehrazi-Raffle, Abhishek Tripathi, Sumanta K. Pal, Clayton S. Lau, Kevin G. Chan, Ali Zhumkhawala, Jonathan Yamzon, Tanya Dorff, Bertram Yuh

**Affiliations:** 1 Division of Urology and Urologic Oncology, Department of Surgery, City of Hope Comprehensive Cancer Center, Duarte, CA 91010, USA; akothari@coh.org (A.K.);; 2 Instituto Nacional de Cancerologia (INCAN), Mexico City 14080, Mexico; rbarraganc@incan.edu.mx; 3 Division of Biostatistics, Department of Computational and Quantitative Medicine, Beckman Research Institute of City of Hope, Duarte, CA 91010, USA; ookunowo@coh.org; 4 Department of Medical Oncology & Experimental Therapeutics, City of Hope Comprehensive Cancer Center, Duarte, CA 91010, USA

**Keywords:** prostate cancer, Latino subpopulations, radical prostatectomy, oncologic outcomes

## Abstract

**Objectives**: Prostate cancer is the most frequently diagnosed cancer among men, with Latino patients experiencing higher cancer-specific mortality compared to non-Hispanic populations. We evaluated prostate cancer outcomes across distinct Latino subpopulations. **Methods**: We retrospectively reviewed a single tertiary comprehensive cancer center database of patients with localized prostate cancer treated with radical prostatectomy from 2003 through 2020. Latino patients were subcategorized as Mexican, South/Central American (SCA), or Caribbean (Puerto Rico and Cuba). Non-Hispanic White (NHW) patients served as the reference group. **Results**: Overall, 7084 patients were included, of which 78% (*n* = 5518) were NHW and 10% (*n* = 679) were Latinos. Compared to NHW, Latino patients were younger at the time of surgery (median 62 vs. 64 years) and had higher median BMI (median 28.8 vs. 27.5 kg/m^2^), median baseline PSA (6.2 vs. 5.5 ng/mL), and D’Amico intermediate- (44% vs. 42%) and high-risk disease rates (16% vs. 13%) (all *p* < 0.001). Among Latino patients, Mexican patients had higher median baseline PSA (6.7 vs. 6.1 vs. 6.1 ng/mL, *p* = 0.005) compared to SCA and Caribbean patients. Survival analysis revealed shorter 5-year (97% vs. 99%) and 10-year (89% vs. 96%) overall survival (OS) rates in Mexican Latinos compared to non-Mexican Latinos (both *p* < 0.05). The 5-year (77% vs. 80% vs. 95%) and 10-year (63% vs. 65% vs. 86%) biochemical recurrence-free survival rates were shorter for Mexican patients compared to SCA and Caribbean patients. On multivariable analysis, SCA subpopulation status was associated with significantly better OS, and pathologic T stage > T2 was associated with worse biochemical recurrence-free survival. **Conclusions**: We identified significant differences in prostate cancer outcomes for different Latino subpopulations, underscoring the importance of population disaggregation in this ethnic group.

## Simple Summary

Prostate cancer is the most frequently diagnosed cancer among men, with Latino patients experiencing worse outcomes and more aggressive disease compared to non-Hispanic/Latino populations. In a retrospective review of 7084 patients with localized prostate cancer treated with radical prostatectomy, we found that Latino patients, compared to non-Hispanic White patients, were significantly younger at the time of surgery and had significantly higher median BMI, median baseline PSA, and D’Amico intermediate- and high-risk disease rates. Latino subpopulation analysis revealed that Mexican patients, compared to South/Central American and Caribbean patients, had higher median baseline PSA at presentation. Survival analysis revealed significantly shorter 5-year and 10-year overall survival and biochemical recurrence-free survival rates in Mexican Latinos. These findings underscore the importance of disaggregating the Latino population in cancer outcomes research.

## 1. Introduction

Prostate cancer is the most common cancer among men in the US [1]. It is also the most commonly diagnosed cancer among Latino men, accounting for 25% of all new cancers diagnosed in this population [2]. While the incidence of prostate cancer among Latino men is 9% lower compared to Non-Hispanic White (NHW) men, the risk of cancer-related mortality is estimated to be 18% higher, raising concerns about racial/ethnic disparities in cancer care [3,4]. The Latino population constitutes the second largest racial/ethnic group in the US, and the largest racial/ethnic group in several US states, including California [5]. Notably, Latinos in the US form a highly heterogeneous ethnic group, given a long history of admixture unique to each Latin American country, over 19 different countries of origin, varying lengths of residency in the US, and differing degrees of acculturation to the US lifestyle [2,4,6,7]. These factors are known to affect the incidence of cancer, adherence to cancer screening recommendations, access to healthcare, and clinical outcomes [7,8,9,10,11].

Yet studies evaluating oncologic outcomes tend to consider Latinos as a single categorical group, which could lead to misrepresented cancer statistics and epidemiological data that undermine the efforts to bridge health disparities [6]. Recent studies have attempted to categorize the Latino population as a conglomerate of multiple different subpopulations (by ancestry or country of origin), providing new insights [9,12]. For example, studies have shown that cancer-related mortality among Latinos of Mexican origin is similar to that for the aggregate group. Conversely, the cancer-specific mortality for Latinos of Cuban and Puerto Rican origin is higher, and for Latinos of Dominican and Central/South American origin, it is the lowest [12]. However, cohort-based studies on prostate cancer have largely been limited to reporting differences in the incidence of prostate cancer, prostate-specific antigen (PSA) levels, or access to treatment across Latino subpopulations [13,14,15], warranting further research evaluating oncologic outcomes and pathological findings.

To the best of our knowledge, no previous study has investigated the clinical characteristics and outcomes of robot-assisted radical prostatectomy among Latino subpopulations. Our institution, a National Cancer Institute (NCI)-designated comprehensive cancer center based in Los Angeles, California, tends to more than 1000 patients with prostate cancer annually, 10% of whom are Latinos. The substantial Latino patient population we have treated over the past several decades gives us a unique opportunity to perform statistically meaningful ethnicity-focused analyses to bridge the gap in our understanding of prostate cancer outcomes in Latino subpopulations. This study aimed to evaluate clinical and pathological characteristics, as well as oncologic outcomes, across distinct Latino subpopulations with localized prostate cancer treated with radical prostatectomy. 

## 2. Materials and Methods

### 2.1. Patient Population

We retrospectively reviewed a single-institution, institutional review board (IRB)-approved (IRB 00149), prospectively maintained database from an NCI-designated cancer center in Southern California, including patients with localized prostate cancer who underwent robot-assisted radical prostatectomy from 2003 to 2020. We included patients with low-, intermediate-, and high-risk localized prostate cancer treated with radical prostatectomy in the upfront or salvage setting. Patients with metastatic disease, who were exclusively treated with radiotherapy/hormone therapy, and those with no self-reported ethnicity were excluded from the analysis. We recorded sociodemographic and clinical characteristics for all patients, including age at surgery, race, ethnicity, body mass index (BMI), American Society of Anesthesiologists (ASA) score, history of prior radiotherapy or hormone therapy, operative time, length of hospital stay, Gleason score, baseline PSA, D’Amico risk score, surgical margins, and rate of lymph node involvement. D’Amico risk groups were defined as follows: low-risk (PSA ≤ 10 ng/mL, Gleason score ≤ 6, and clinical stage T1–T2a), intermediate-risk (PSA 10–20 ng/mL, Gleason score 7, or clinical stage T2b), and high-risk (PSA > 20 ng/mL, Gleason score 8–10, or clinical stage ≥ T2c). Patients were categorized based on self-reported ethnicity as Hispanic or Latino, NHW, Black or African American, and Asian/American Indian, Alaskan Native (AIAN)/Native Hawaiian and other Pacific Islander (NHOPI). Latino patients were further subcategorized as Mexican, South/Central American (SCA), or Caribbean (Puerto Rico and Cuba). NHW patients served as the reference group for intergroup comparisons. Patients without an identifiable country of origin (Spanish not otherwise specified or NOS subgroup) were excluded from subgroup survival analysis. The distribution of patients across subgroups is shown in Supplementary Figure S1.

### 2.2. Statistical Analysis

Descriptive statistics were used to summarize clinical and pathological variables. Categorical data are reported as counts and percentages. Continuous data are reported as medians and interquartile ranges (IQRs). Comparisons between categorical variables were made using Fisher’s exact test or Chi-squared test, as appropriate. Comparisons between continuous variables were performed using the Kruskal–Wallis rank sum test. Overall survival (OS) was estimated from the date of surgery to the date of death from any cause. Biochemical recurrence-free survival (BCRFS) was defined as the time from surgery to the date of confirmed biochemical recurrence, defined as a PSA ≥ 0.2 ng/mL on two consecutive measurements following radical prostatectomy. Patients were censored at the last known follow-up if their vital status was unknown. Kaplan–Meier analysis and log-rank test compared OS and BCRFS between Latino subpopulations. Univariable and multivariable Cox proportional hazards regression analyses were performed to evaluate independent predictors of OS and BCRFS. Variables included ethnicity, age at surgery, BMI, baseline PSA, prior hormone therapy status, D’Amico risk score, and pathologic tumor stage. Variables with significance in the univariable model were included in the multivariable model. Results are reported as hazard ratios (HRs) with 95% confidence intervals (CIs). Additionally, a logistic regression model was constructed to identify variables that predict high-risk disease at initial diagnosis among Hispanic/Latino patients. Results are reported as odds ratios (ORs) with 95% CI. Data management and statistical analyses were conducted using SAS version 9.4 (SAS Institute Inc., Cary, NC, USA) and R version 4.3.0 (R Foundation for Statistical Computing). *p*-values < 0.05 were considered statistically significant.

## 3. Results

A total of 7100 patients with localized prostate cancer treated with prostatectomy from 2003 to 2020 were identified, of which 7084 patients met the inclusion criteria. Among these, 78% of patients (*n* = 5518) were categorized as NHW, 10% (*n* = 679) were Hispanic/Latinos, 7% (*n* = 525) were Asian/AIAN/NHOPI, and 5% (*n* = 362) were Black/African American. Hispanic/Latino (62 years, IQR 57–68) and Black/African American (61.7 years, IQR 56–66) patients were more likely to be younger at the time of surgery compared to NHW (64 years, IQR 58–69) patients (*p* < 0.001). Baseline PSA (ng/mL) was more likely to be higher among Asian/AIAN/NHOPI (6.9, IQR 4.9–10.0) patients, followed by Black/African American (6.6, IQR 4.9–9.9) and Hispanic/Latino (6.2, IQR 4.7–9.4) patients (*p* < 0.001). Compared to NHW, Hispanic/Latino patients had higher rates of D’Amico intermediate- (44% vs. 42%) and high-risk disease (16% vs. 13%) (all *p* < 0.001), respectively. Pathology Gleason scores 3 + 3 and 3 + 4 were more likely among NHW (30%) and Black/African American patients (55%), respectively (*p* < 0.001). Asian/AIAN/NHOPI (30%) and Hispanic/Latino (26%) patients were more likely to show positive surgical margins (*p* = 0.03) compared to other ethnicities. Hispanic/Latino (7%) and Asian/AIAN/NHOPI (7%) patients were more likely to show at least one positive lymph node compared to NHW (4%) patients (*p* < 0.001). Compared to other ethnicities, NHW (80%) patients were more likely to show pathologic stage 2 (pT2) disease, while Hispanic/Latino (13%) patients were more likely to show pathologic stage 3b (pT3b) disease (all, *p* < 0.001). Demographic, clinical, and pathologic characteristics for all patients can be found in Table 1 and Table 2. Among Latino patients, 45% (*n* = 304) were Mexican, 34% (*n* = 233) were Spanish NOS/other, 11% (*n* = 75) were SCA, and 3% (*n* = 22) were Caribbean. Compared to SCA and Caribbean patients, Mexican patients had higher median baseline PSA (ng/mL) (6.7 vs. 6.1 vs. 6.1, *p* = 0.005) and longer median operative times (min) (188 vs. 176 vs. 173, *p* = 0.02). Demographic, clinical, and pathologic characteristics for Hispanic/Latino patients can be found in Table 3 and Table 4.

**Figure 1:**
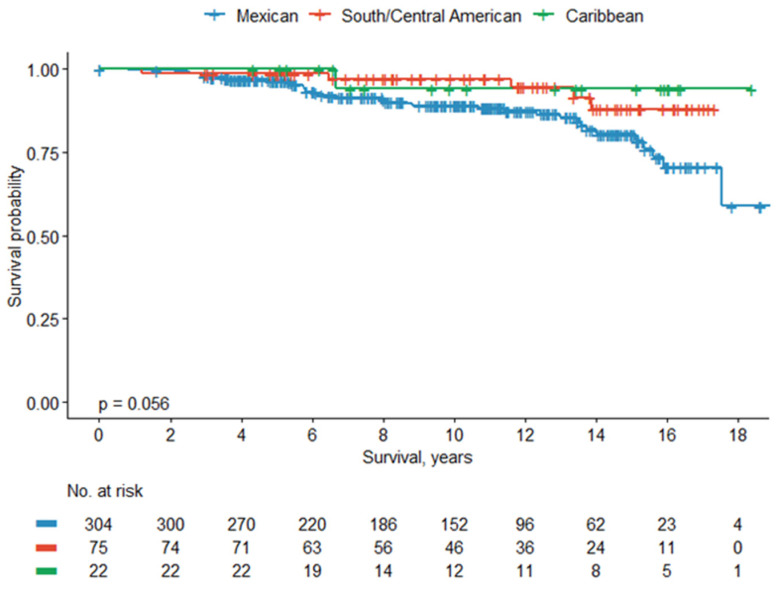
Overall survival between Mexican vs. South/Central American vs. Caribbean patients.

**Figure 2:**
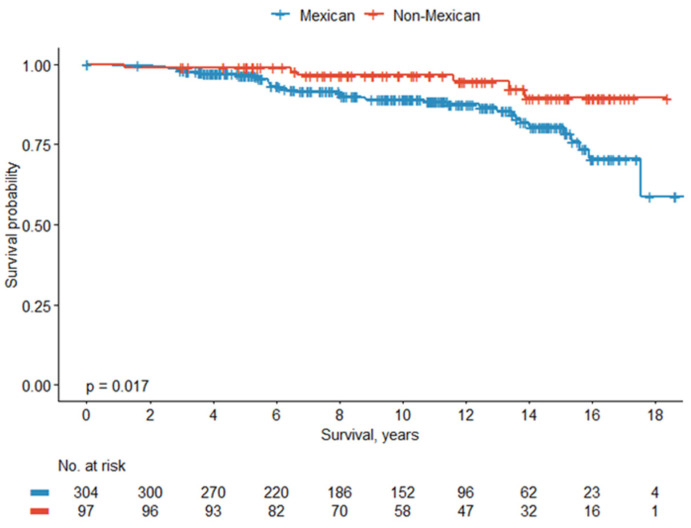
Overall survival between Mexican vs. non-Mexican patients.

Subgroup survival analysis of Hispanic/Latino subpopulations (*n* = 401) revealed a shorter 5-year (97%) and 10-year (89%) OS rate in Mexican patients compared to SCA (99% and 97%, respectively) and Caribbean (100% and 94%, respectively) (*p* = 0.056). Subgroup analysis comparing Mexican vs. non-Mexican Latino subpopulations revealed a shorter 5-year (97%) and 10-year (89%) OS rate for Mexican patients compared to non-Mexican patients (99% and 96%, respectively) (*p* = 0.01). Kaplan–Meier curves showing the overall survival rate between Hispanic/Latino subpopulations can be found in Figure 1 and Figure 2.

**Figure 3:**
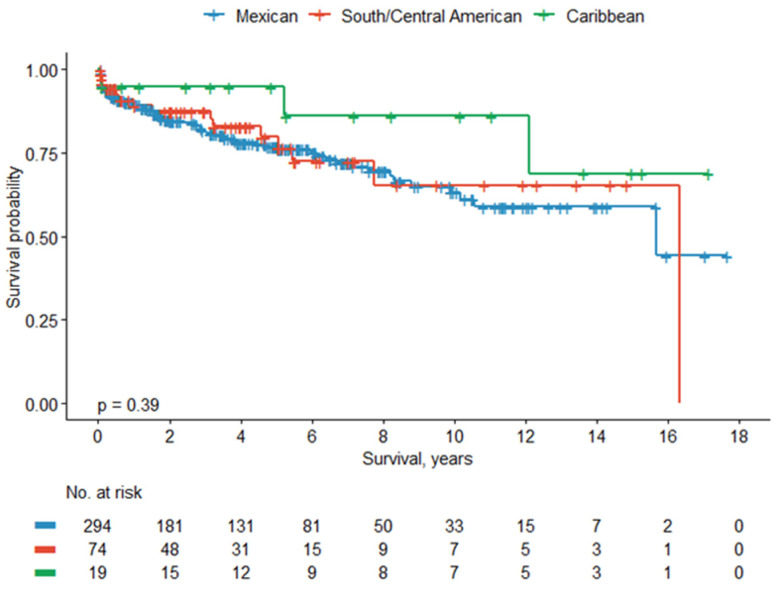
Biochemical recurrence-free survival between Hispanic/Latino subpopulations.

Analysis of BCRFS among Hispanic/Latino subpopulations (*n* = 387) similarly demonstrated lower BCRFS rates in Mexican patients across all timepoints compared to SCA and Caribbean patients. The 5- and 10-year BCRFS rates were 77% and 63% for Mexican patients, 80% and 65% for SCA patients, and 95% and 86% for Caribbean patients, respectively. However, this was not statistically significant (*p* = 0.39). Kaplan–Meier curves showing BCRFS for Latino subpopulations are shown in Figure 3.

On multivariable analysis for OS, SCA subpopulation status was independently associated with significantly better OS (HR 0.33, 95% CI 0.12–0.95, *p* = 0.04). On multivariable analysis for BCRFS, pathologic T stage > T2 was independently associated with significantly worse outcomes (HR 4.32, 95% CI 2.49–7.48, *p* < 0.001). Full results of univariable and multivariable Cox regression analyses are presented in Supplementary Tables S1 and S2.

Logistic regression analysis showed that a higher baseline PSA (OR 1.04, 95% CI 1.01–1.06, *p* = 0.007) and prior hormone therapy (OR 3.04, 95% CI 1.15–7.46, *p* = 0.01) were independently associated with high-risk disease at diagnosis among Hispanic/Latino patients. Full logistic regression analysis is shown in Supplementary Table S3.

## 4. Discussion

Studies on oncologic outcomes frequently consider the entire Latino population as a single cohort without acknowledging the substantial differences among the subpopulations that constitute it. We conducted a retrospective study evaluating the clinical and pathologic characteristics, as well as oncologic outcomes of robot-assisted radical prostatectomy across Latino subpopulations. Our analysis revealed statistically significant differences in OS, BCRFS, and baseline PSA levels across subpopulations.

A study performed by Del Pino et al. used the National Cancer Database to compare diagnostic and treatment data among Latino patients with prostate cancer, categorized by country of origin. Echoing our findings, this study found that Mexican patients had the highest baseline median PSA (7.2 ng/mL), followed by SCA (6.9 ng/mL), Cuban (6.6 ng/mL), and Puerto Rican (6.4 ng/mL) Latinos (*p* < 0.001) [13]. Reports on mortality/survival among Latino subpopulations with prostate cancer are highly variable. In Del Pino’s study, the Dominican and SCA subpopulations showed improved OS, Cubans exhibited worse OS, and Puerto Ricans showed comparable OS [13]. Similarly, one study found that prostate cancer-specific mortality after radical prostatectomy was the highest among Puerto Rican patients, followed by SCA, Cuban, and Mexican patients [15]. Meanwhile, another study found that both Mexican and Puerto Rican subpopulations had significantly higher prostate cancer-specific mortality compared to SCA and Cuban subpopulations [16]. National mortality data from 1999 to 2020 similarly showed that Mexican-origin men generally have cancer mortality rates similar to or higher than the aggregate Hispanic population, whereas Puerto Rican men have some of the highest prostate cancer-specific mortality rates among Latino subgroups [17]. We found that the Mexican subpopulation in our cohort had the lowest 5-year and 10-year OS and BCRFS rates compared to other Latino subpopulations. Various biological, cultural, psychosocial, socioeconomic, or clinical/systemic factors, known to influence cancer diagnosis, treatment, and survivorship among Latinos in the US, could explain this observation [18]. Firstly, Mexican patients in our study population had the highest median PSA at baseline, which is a biological risk factor associated with more aggressive disease profiles [19]. Secondly, Mexican patients with prostate cancer tend to have longer diagnosis-to-treatment times than other Latino subpopulations [20]. This has been found to be associated with worse predicted 5-year and 10-year OS rates, particularly among patients with intermediate- and high-risk prostate cancer [21]. Thirdly, Mexican patients are significantly less likely to seek treatment for localized prostate cancer compared to Cuban, SCA, or Puerto Rican patients, which could affect treatment timelines and, consequently, oncologic outcomes [14]. In this context, our finding of shorter OS and BCRFS among Mexican-origin men after radical prostatectomy highlights the value of subpopulation-specific analyses, as aggregate Hispanic data may mask clinically meaningful disparities. Addressing the drivers of these differences, including baseline disease characteristics, delays in diagnosis and treatment, and systemic barriers to timely, guideline-concordant care, will be critical to improving prostate cancer outcomes across diverse Latino communities. For example, both delayed treatment and poor treatment-seeking are often a culmination of cultural, socioeconomic, and systemic/clinical factors that are known to disproportionately impact Latinos and other underserved populations in the US. Notably, Latinos in the US are more likely than other ethnic groups to delay cancer treatment due to lack of insurance, insufficient coverage, or financial toxicity [22,23,24]. Among survivors of cancer from different Latino subpopulations, Mexican, SCA, and Cuban Latinos, but not Puerto Rican and Dominican Latinos, have significantly greater odds of healthcare unaffordability compared to NHW survivors [23]. Other factors, such as nativity (foreign- vs. US-born), further influence insurance status, with foreign-born Latinos being more likely to be uninsured than US-born Latinos [22]. Cultural factors may also affect treatment-related decision-making among Latino patients with prostate cancer [25,26]. As such, individuals’ cultural perception varies by nativity, immigrant generation, and degree of acculturation, adding to the complexity of factors that affect prostate cancer outcomes among different subpopulations [27]. Additionally, Latino patients with prostate cancer may experience a lack of access to continuous and well-coordinated guideline-concordant screening and care, which may further vary by country of origin [28,29].

Overall, our study adds to the limited number of investigations of prostate cancer outcomes in Latino subpopulations, providing more evidence to support the disaggregation of data on Latino patients and the need for granular analyses to extract finer differences in patient outcomes by ethnic subpopulation. Moreover, our study focuses on a unique subpopulation of patients with prostate cancer—those who underwent robot-assisted radical prostatectomy. Previous studies have focused either on an all-encompassing population of patients with prostate cancer [13,14,16] or on patients who underwent any type of radical prostatectomy for prostate cancer [15]. To the best of our knowledge, there are no studies that investigate the outcomes of robot-assisted radical prostatectomy in a disaggregated Latino population.

This study also has several important limitations. Due to the retrospective nature of this study, data on specific sociodemographic characteristics (such as socioeconomic status, degree of acculturation, length of residency, linguistic barriers, or educational status) and clinical characteristics (such as pelvic visceral fat volume or adiposity), which are likely to affect clinical outcomes among Latinos, were not available for analysis. Additionally, comprehensive genetic profiling was not available. Furthermore, the multidimensionality of factors influencing the heterogeneity among Latino subpopulations makes it challenging to establish causality for varying clinical outcomes. Comparative analyses between Latino subgroups, involving sociodemographic factors such as socioeconomic status, nativity, degree of acculturation, length of residency in the US, insurance status, and access to guideline-concordant care, were beyond the scope of the present report. These factors are particularly important to consider in investigations of the Latino population because of the so-called “Hispanic/Latino health paradox”, a collection of phenomena which include observations that (1) Latinos in the US, despite being more likely to be at a socioeconomic disadvantage, tend to have comparable or improved health outcomes when compared to other racial/ethnic groups, including NHWs; and (2) immigrant Latinos (especially recent immigrants) have better health outcomes than US-born Latinos [30,31,32]. Detailed sociodemographic data must be collected and analyzed to determine if this paradox is observed in our cohort. Thus, future studies exploring these variables and their impact on clinical outcomes of different Latino subpopulations are warranted. The relatively small sample sizes of the Caribbean and South/Central American subgroups reduce the statistical power of subgroup survival analysis. Furthermore, a substantial number of patients could not be assigned to a specific subgroup and were therefore excluded from survival analysis, as arbitrary subgroup assignment could introduce misclassification bias. In addition, our results might be affected by selection bias by including Hispanic/Latino patients living in Southern California. As such, the results of subgroup comparisons should be interpreted with caution.

Importantly, our study uses a classification system that disaggregates the Latino population in the US by country of origin. It does not consider admixture within Latino subpopulations. For example, nearly 93% of the Latinos of Mexican origin have mixed Indigenous American (North and South; 50–60%), European (40–45%), and African (2–3%) ancestries [33,34]. Puerto Rican Latinos, on the other hand, have >65% European ancestry, followed by African ancestry (~18%), and Indigenous ancestry (~14%) [35]. Notably, the proportion of different genetic ancestries in the Latino population affects prostate cancer risk as well as outcomes. Du et al. found that a 0.1-point increase in the percentage of Indigenous American ancestry was associated with a 16% decrease in prostate cancer risk in global ancestry analysis [36]. Meanwhile, local ancestry analysis of prostate cancer risk regions in the genome revealed a significant association of risk with African ancestry [36]. Irizarry-Ramírez et al. found that Puerto Rican men with one or more prostate cancer risk alleles were more likely to have a higher proportion of West African ancestry [37]. Other studies have noted a positive association between African ancestry and risk of prostate cancer incidence at a younger age [38,39]. Thus, it is likely that admixture affected the patient outcomes in our study, but detailed studies are needed to confirm its contribution.

## 5. Conclusions

Our study revealed that Hispanic/Latino subpopulations with localized prostate cancer exhibit significant differences in clinical presentation and oncologic outcomes. We noted a trend for improved outcomes for SCA patients, as opposed to Mexican and Caribbean patients. Moreover, our multivariable analysis revealed that higher pathologic tumor stages significantly predicted worse BCRFS, underscoring the importance of strict surveillance. Furthermore, higher baseline PSA and a history of hormone therapy were independently associated with high-risk disease at diagnosis among Latino patients, highlighting potential targets for earlier intervention and risk stratification in this population. Our findings highlight the significance of population-based analysis to improve our understanding of the clinical outcomes in localized prostate cancer. Prospective studies incorporating sociodemographic determinants, genetic ancestry, and healthcare access metrics are warranted to further elucidate the drivers of disparities and to inform culturally tailored strategies.

## Figures and Tables

**Table 1:** Baseline demographic and clinical characteristics for all patients.

		Race/Ethnicity				
Variable	Overall *N* = 7084 ^1^	Asian/NHOPI/AIAN *n* = 525 ^1^	Black or African American *n* = 362 ^1^	Hispanic or Latino *n* = 679 ^1^	Non-Hispanic White *n* = 5518 ^1^	*p*-Value ^2^
Follow-up time, years						<0.001
Median (IQR)	13.0 (8.9, 15.9)	11.8 (6.7, 15.2)	11.8 (7.6, 15.1)	11.5 (7.3, 15.1)	13.3 (9.5, 16.0)	
Missing	9	0	0	0	9	
Age at surgery, years						<0.001
Median (IQR)	64.0 (58.0, 69.0)	66.0 (61.0, 70.6)	61.7 (56.0, 66.0)	62.0 (57.0, 68.0)	64.0 (58.0, 69.0)	
Missing	4	2	0	0	2	
BMI, kg/m^2^						<0.001
Median (IQR)	27.5 (25.1, 30.3)	25.4 (23.4, 27.9)	28.9 (26.3, 32.5)	28.8 (26.1, 31.9)	27.5 (25.2, 30.3)	
Missing	161	27	9	22	103	
Prior radiation treatment, *n* (%)	78 (1%)	3 (1%)	9 (2%)	7 (1%)	59 (1%)	0.07
Prior hormone treatment, *n* (%)	542 (8%)	66 (13%)	33 (9%)	62 (9%)	381 (7%)	<0.001
Prior chemotherapy treatment, *n* (%)	11 (0%)	2 (0%)	0 (0%)	0 (0%)	9 (0%)	0.3
Prior other treatment, *n* (%)	234 (3%)	19 (4%)	12 (3%)	25 (4%)	178 (3%)	>0.9
Operative time, minutes						<0.001
Median (IQR)	174.0 (152.0, 204.0)	167.0 (150.0, 198.0)	190.0 (167.0, 222.0)	182.0 (158.0, 216.0)	173.0 (150.0, 202.0)	
Missing	68	14	7	10	37	
ASA, *n* (%)						0.2
I	32 (1%)	4 (1%)	2 (1%)	2 (0%)	24 (1%)	
II	1743 (47%)	134 (45%)	94 (40%)	181 (43%)	1334 (48%)	
III	1828 (49%)	147 (50%)	130 (55%)	227 (54%)	1324 (47%)	
IV	143 (3%)	11 (4%)	11 (4%)	12 (3%)	109 (4%)	
Missing	3338	229	125	257	2727	
Baseline PSA						<0.001
Median (IQR)	5.7 (4.4, 8.2)	6.9 (4.9, 10.0)	6.6 (4.9, 9.9)	6.2 (4.7, 9.4)	5.5 (4.3, 7.8)	
Missing	67	6	4	12	45	
D’Amico Risk, *n* (%)						<0.001
Low	2669 (43%)	158 (38%)	96 (34%)	215 (40%)	2200 (45%)	
Intermediate	2611 (42%)	176 (42%)	132 (47%)	238 (44%)	2065 (42%)	
High	892 (15%)	86 (20%)	53 (19%)	85 (16%)	668 (13%)	
Missing	912	105	81	141	585	
EBL, mL						<0.001
Median (IQR)	200.0 (150.0, 300.0)	150.0 (100.0, 250.0)	200.0 (150.0, 300.0)	200.0 (150.0, 300.0)	200.0 (150.0, 300.0)	
Missing	632	71	64	84	413	
Length of hospital stay, days						0.2
Median (IQR)	1.0 (1.0, 2.0)	1.0 (1.0, 2.0)	1.0 (1.0, 2.0)	1.0 (1.0, 2.0)	1.0 (1.0, 2.0)	
Missing	87	15	6	13	53	

^1^ *n* (%). ^2^ Kruskal–Wallis rank sum test; Fisher’s exact test; Pearson’s Chi-squared test. Abbreviations: AIAN, American Indian or Alaskan Native; ASA, American Society of Anesthesiologists; BMI, body mass index; IQR, inter-quartile range; NHOPI, Native Hawaiian and Other Pacific Islander.

**Table 2:** Pathologic data for all patients.

	Race/Ethnicity					
Variable	Overall *N* = 7084 ^1^	Asian/NHOPI/AIAN *n* = 525 ^1^	Black or African American *n* = 362 ^1^	Hispanic or Latino *n* = 679 ^1^	Non-Hispanic White *n* = 5518 ^1^	*p*-Value ^2^
Pathology Gleason, *n* (%) *						<0.001
<3 + 3	19 (0%)	1 (0%)	3 (1%)	1 (0%)	14 (0%)	
3 + 3	1898 (29%)	103 (22%)	59 (18%)	167 (28%)	1569 (30%)	
3 + 4	3019 (45%)	196 (42%)	185 (55%)	266 (44%)	2372 (45%)	
4 + 3	1171 (18%)	113 (24%)	64 (18%)	127 (20%)	867 (17%)	
4 + 4	201 (3%)	24 (5%)	12 (4%)	16 (3%)	149 (3%)	
>4 + 4	341 (5%)	33 (7%)	14 (4%)	29 (5%)	265 (5%)	
Missing	435	55	25	73	282	
Margins, *n* (%)						0.035
Negative	5335 (76%)	362 (70%)	267 (75%)	499 (74%)	4207 (77%)	
Positive	1692 (24%)	154 (30%)	90 (25%)	173 (26%)	1275 (23%)	
Unknown	3 (0%)	0 (0%)	0 (0%)	0 (0%)	3 (0%)	
Missing	54	9	5	7	33	
Lymph nodes removed						<0.001
Median (IQR)	3.0 (0.0, 10.0)	4.0 (0.0, 12.0)	5.0 (0.0, 13.0)	4.0 (0.0, 12.0)	3.0 (0.0, 9.0)	
Missing	46	6	4	7	29	
Positive lymph node(s), categorized, *n* (%)						<0.001
0	6719 (95%)	484 (93%)	331 (92%)	628 (93%)	5276 (96%)	
1–5	281 (4%)	32 (7%)	23 (6%)	39 (7%)	187 (4%)	
6–10	27 (1%)	2 (0%)	2 (1%)	3 (0%)	20 (0%)	
>10	12 (0%)	2 (0%)	2 (1%)	2 (0%)	6 (0%)	
Missing	45	5	4	7	29	
Pathology T-stage, *n* (%)						<0.001
T2	5436 (79%)	373 (74%)	259 (73%)	499 (76%)	4305 (80%)	
T3a	870 (12%)	77 (15%)	52 (15%)	71 (11%)	670 (12%)	
T3b	592 (9%)	49 (10%)	44 (11%)	84 (13%)	415 (8%)	
T4	15 (0%)	3 (1%)	2 (1%)	2 (0%)	8 (0%)	
TX	7 (0%)	1 (0%)	0 (0%)	1 (0%)	5 (0%)	
Missing	164	22	5	22	115	

^1^ *n* (%). ^2^ Kruskal–Wallis rank sum test; Fisher’s exact test; Pearson’s Chi-squared test. Abbreviations: AIAN, American Indian or Alaskan Native; IQR, inter-quartile range; NHOPI, Native Hawaiian and Other Pacific Islander. * >4 + 4 Gleason includes: 4 + 5, 5 + 4, 5 + 5, 3 + 5, 5 + 3.

**Table 3:** Patient demographic and clinical characteristics for Hispanic patients.

		Hispanic Subgroups					
Variable	Overall *N* = 679 ^1^	Mexican *n* = 304 ^1^	South/Central American *n* = 75 ^1^	Caribbean *n* = 22 ^1^	Spanish, NOS/Other *n* = 233 ^1^	Unknown *n* = 45 ^1^	*p*-Value ^2^
Follow-up time, years							<0.001
Median (IQR)	11.5 (7.3, 15.1)	10.0 (5.6, 13.3)	11.9 (8.0, 14.7)	11.6 (6.7, 15.9)	14.2 (10.6, 16.6)	12.6 (9.7, 14.4)	
Age at surgery, years							0.14
Median (IQR)	62.0 (57.0, 68.0)	62.4 (57.0, 68.0)	62.0 (57.0, 67.0)	61.5 (58.0, 65.0)	62.0 (57.0, 68.0)	57.0 (54.0, 64.6)	
BMI, kg/m^2^							>0.9
Median (IQR)	28.8 (26.1, 31.9)	28.7 (26.1, 31.7)	28.8 (26.6, 30.9)	30.0 (24.3, 32.3)	28.9 (26.0, 32.1)	28.9 (26.6, 31.6)	
Missing	22	12	3	2	1	4	
Prior radiation treatment, *n* (%)	7 (1%)	4 (1%)	1 (1%)	0 (0%)	2 (1%)	0 (0%)	0.9
Prior hormone treatment, *n* (%)	62 (9%)	23 (8%)	10 (13%)	2 (9%)	24 (10%)	3 (7%)	0.5
Prior other treatment, *n* (%)	25 (4%)	7 (2%)	5 (7%)	0 (0%)	11 (5%)	2 (4%)	0.2
Operative time, minutes							0.02
Median (IQR)	182.0 (158.0, 216.0)	188.0 (163.5, 222.0)	176.0 (150.0, 210.0)	173.0 (149.0, 214.0)	180.0 (157.0, 212.0)	169.0 (147.0, 201.0)	
Missing	10	8	1	1	0	0	
ASA, *n* (%)							0.5
I	2 (0%)	1 (0%)	0 (0%)	0 (0%)	0 (0%)	1 (3%)	
II	181 (43%)	90 (39%)	21 (44%)	6 (46%)	45 (46%)	19 (58%)	
III	227 (54%)	134 (58%)	25 (52%)	7 (54%)	49 (51%)	12 (36%)	
IV	12 (3%)	6 (3%)	2 (4%)	0 (0%)	3 (3%)	1 (3%)	
Missing	257	73	27	9	136	12	
Baseline PSA							0.005
Median (IQR)	6.2 (4.7, 9.4)	6.7 (5.0, 10.3)	6.1 (4.7, 11.1)	6.1 (4.4, 9.2)	6.0 (4.6, 8.4)	5.2 (4.2, 7.0)	
Missing	12	4	2	0	4	2	
D’Amico Risk, *n* (%)							0.06
Low	215 (40%)	70 (33%)	18 (31%)	6 (38%)	103 (49%)	18 (46%)	
Intermediate	238 (44%)	107 (50%)	28 (48%)	7 (44%)	80 (38%)	16 (41%)	
High	85 (16%)	37 (17%)	12 (21%)	3 (18%)	28 (13%)	5 (13%)	
Missing	141	90	17	6	22	6	
EBL, mL							0.2
Median (IQR)	200.0 (150.0, 300.0)	200.0 (150.0, 300.0)	200.0 (150.0, 250.0)	200.0 (150.0, 250.0)	200.0 (150.0, 300.0)	175.0 (125.0, 275.0)	
Missing	84	49	10	5	15	5	
Length of hospital stay, days							<0.001
Median (IQR)	1.0 (1.0, 2.0)	1.0 (1.0, 1.0)	1.0 (1.0, 2.0)	1.0 (1.0, 1.0)	1.0 (1.0, 2.0)	1.0 (1.0, 2.0)	
Missing	13	10	2	1	0	0	

^1^ *n* (%). ^2^ Kruskal–Wallis rank sum test; Fisher’s exact test; Pearson’s Chi-squared test. Abbreviations: ASA, American Society of Anesthesiologists; BMI, body mass index; IQR, inter-quartile range; NOS, not otherwise specified. Spanish NOS/Other Hispanic sub-group includes Spanish, NOS; Other specified Spanish; Spanish surname only. Caribbean sub-group includes Puerto Ricans and Cubans.

**Table 4:** Pathologic data for Hispanic patients.

		Hispanic Subgroups					
Variable	Overall *N* = 679 ^1^	Mexican *n* = 304 ^1^	South/Central American *n* = 75 ^1^	Caribbean *n* = 22 ^1^	Spanish, NOS/Other *n* = 233 ^1^	Unknown *n* = 45 ^1^	*p*-Value ^2^
Pathology Gleason, *n* (%) *							0.5
<3 + 3	1 (0%)	0 (0%)	0 (0%)	0 (0%)	1 (0%)	0 (0%)	
3 + 3	167 (28%)	55 (22%)	19 (28%)	2 (10%)	75 (34%)	16 (36%)	
3 + 4	266 (44%)	115 (46%)	32 (46%)	12 (60%)	91 (41%)	16 (36%)	
4 + 3	127 (20%)	58 (22%)	14 (20%)	4 (20%)	42 (20%)	9 (21%)	
4 + 4	16 (3%)	7 (3%)	2 (3%)	1 (5%)	5 (2%)	1 (2%)	
>4 + 4	29 (5%)	17 (7%)	2 (3%)	1 (5%)	7 (3%)	2 (5%)	
Missing	73	52	6	2	12	1	
Pathology T-stage, *n* (%)							0.6
T2	499 (76%)	203 (71%)	57 (77%)	14 (70%)	188 (82%)	37 (84%)	
T3a	71 (11%)	39 (13%)	8 (11%)	3 (15%)	18 (8%)	3 (7%)	
T3b	84 (13%)	44 (15%)	9 (12%)	3 (15%)	24 (10%)	4 (9%)	
T4	2 (0%)	2 (1%)	0 (0%)	0 (0%)	0 (0%)	0 (0%)	
TX	1 (0%)	1 (0%)	0 (0%)	0 (0%)	0 (0%)	0 (0%)	
Missing	22	15	1	2	3	1	
Margins, *n* (%)							0.3
Negative	499 (74%)	211 (71%)	59 (79%)	18 (86%)	175 (75%)	36 (82%)	
Positive	173 (26%)	88 (29%)	16 (21%)	3 (14%)	58 (25%)	8 (18%)	
Unknown	0 (0%)	0 (0%)	0 (0%)	0 (0%)	0 (0%)	0 (0%)	
Missing	7	5	0	1	0	1	
Lymph nodes removed							<0.001
Median (IQR)	4.0 (0.0, 12.0)	5.0 (0.0, 16.0)	3.0 (0.0, 11.0)	5.0 (0.0, 8.0)	3.0 (0.0, 7.0)	1.0 (0.0, 8.0)	
Missing	7	7	0	0	0	0	
Positive lymph node(s), categorized, *n* (%)							0.3
0	628 (93%)	269 (91%)	72 (96%)	19 (86%)	226 (97%)	42 (93%)	
1–5	39 (7%)	24 (8%)	3 (4%)	3 (14%)	6 (3%)	3 (7%)	
6–10	3 (0%)	3 (1%)	0 (0%)	0 (0%)	0 (0%)	0 (0%)	
>10	2 (0%)	1 (0%)	0 (0%)	0 (0%)	1 (0%)	0 (0%)	
Missing	7	7	0	0	0	0	

^1^ *n* (%). ^2^ Pearson’s Chi-squared test; Fisher’s exact test; Kruskal–Wallis rank sum test. Abbreviations: IQR, inter-quartile range; NOS, not otherwise specified. Spanish NOS/Other Hispanic sub-group includes Spanish, NOS; Other specified Spanish; Spanish surname only. Caribbean sub-group includes Puerto Ricans and Cubans. * >4 + 4 Gleason includes: 4 + 5, 5 + 4, 5 + 5, 3 + 5, 5 + 3.

## Data Availability

The datasets generated and/or analyzed during the current study are not publicly available due to institutional policies but are available from the corresponding author on reasonable request.

## Supplementary Materials

The following supporting information can be downloaded at: https://www.mdpi.com/article/10.3390/cancers18061035/s1, Figure S1: Distribution of patients across ethnic groups; Table S1: Univariable and multivariable hazards model for overall survival; Table S2: Univariable and multivariable hazards model for biochemical recurrence-free survival; Table S3: Logistic regression models predicting high-risk disease at initial diagnosis among Hispanic/Latino patients.

## Abbreviations

The following abbreviations are used in this manuscript:

AIAN	American Indian and Alaskan Native
ASA	American Society of Anesthesiologists
BCRFS	Biochemical recurrence-free survival
BMI	Body mass index
IQR	Interquartile range
IRB	Institutional review board
NCI	National Cancer Institute
NHOPI	Native Hawaiian and Other Pacific Islander
NHW	Non-Hispanic White
NOS	Not otherwise specified
OS	Overall survival
PSA	Prostate-specific antigen
SCA	South/Central American
US	United States
