# Supplementary material for: Disparities in Outcomes in Latino Subpopulations with Localized Prostate Cancer Undergoing Radical Prostatectomy: A Population-Based Analysis
Source: Cancers (Basel). 2026 Mar 23;18(6):1035. doi: 10.3390/cancers18061035 (PMC13024825; doi:10.3390/cancers18061035)
Supplement: Supplementary file 1 [file cancers-18-01035-s001.zip › cancers-4177719-supplementary.pdf]

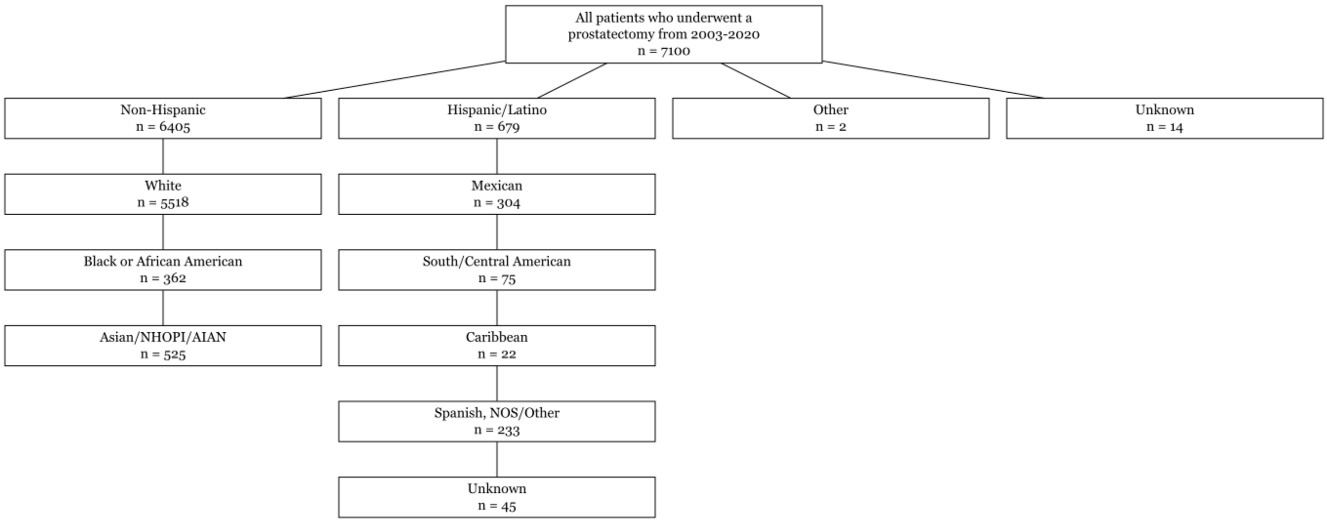

Supplementary Figure S1. Distribution of patients across ethnic groups.

Supplementary Table S1. Univariable and multivariable hazards model for overall survival.

	Univariable Analysis		Multivariable Analysis	
**Characteristic**	**HR (95% CI)**	**P-value**	**HR (95% CI)**	**P-value**
Ethnicity				
Mexican	—		—	
South/Central American	0.40 (0.16, 1.02)	0.05	0.33 (0.12, 0.95)	**0.04**
Caribbean	0.25 (0.03, 1.84)	0.17	0.29 (0.04, 2.18)	0.23
Age at surgery, years	1.04 (1.00, 1.08)	0.05	1.04 (0.99, 1.09)	0.08
BMI, kg/m²	1.02 (0.96, 1.08)	0.47	1.02 (0.95, 1.10)	0.59
Baseline PSA	1.01 (1.00, 1.03)	0.10	1.00 (0.98, 1.03)	0.84
Prior hormone treatment	1.99 (0.93, 4.26)	0.07	2.04 (0.84, 4.98)	0.12
D'Amico Risk	1.61 (1.04, 2.50)	**0.03**	1.17 (0.69, 1.99)	0.57
Pathology T-stage				
T2	—		—	
>T2	2.75 (1.53, 4.96)	**<0.001**	1.87 (0.92, 3.78)	0.08

Abbreviations: HR, Hazard Ratio; CI, Confidence Interval.

Supplementary Table S2. Univariable and multivariable hazards model for biochemical recurrence-free survival.

	Univariable Analysis		Multivariable Analysis	
**Characteristic**	**HR (95% CI)**	**P-value**	**HR (95% CI)**	**P-value**
Ethnicity				
Mexican	—		—	
South/Central American	0.92 (0.53, 1.61)	0.77	0.77 (0.38, 1.59)	0.49
Caribbean	0.45 (0.14, 1.45)	0.18	0.49 (0.12, 2.03)	0.32
Age at surgery, years	0.99 (0.96, 1.02)	0.60	0.97 (0.94, 1.01)	0.16
BMI, kg/m²	1.03 (0.99, 1.08)	0.13	1.02 (0.96, 1.08)	0.54
Baseline PSA	1.02 (1.01, 1.03)	**<0.001**	1.01 (1.00, 1.03)	0.15
Prior hormone treatment	2.10 (1.18, 3.74)	**0.01**	0.88 (0.42, 1.84)	0.73
D'Amico Risk	2.00 (1.37, 2.91)	**<0.001**	1.45 (0.89, 2.36)	0.14
Pathology T-stage				
T2	—		—	
>T2	4.63 (2.98, 7.19)	**<0.001**	4.32 (2.49, 7.48)	**<0.001**

Abbreviations: HR, Hazard Ratio; CI, Confidence Interval.

Supplementary Table S3. Logistic regression models predicting high-risk disease at initial diagnosis among Hispanic/Latino patients.

**Characteristic**	**OR (95% CI)**	**P-value**
Ethnicity		
Mexican	—	
South/Central American	1.09 (0.47, 2.30)	0.8
Caribbean	1.75 (0.48, 5.03)	0.3
Age at surgery, years	1.04 (0.99, 1.09)	0.14
BMI, kg/m²	1.05 (0.97, 1.12)	0.2
Baseline PSA	1.04 (1.01, 1.06)	**0.007**
Prior hormone treatment	3.04 (1.15, 7.46)	**0.01**

Abbreviations: OR, Odds Ratio; CI, Confidence Interval.
